# Supplementary material for: Robust but weak winter atmospheric circulation response to future Arctic sea ice loss
Source: Nat Commun. 2022 Feb 7;13:727. doi: 10.1038/s41467-022-28283-y (PMC8821642; doi:10.1038/s41467-022-28283-y)
Supplement: Supplementary file 1 — Supplementary Information [file 41467_2022_28283_MOESM1_ESM.pdf]

Supplementary Information for

Robust but weak winter atmospheric circulation response to future Arctic sea ice loss

D. M. Smith[1], R. Eade[1], M. B. Andrews[1], H. Ayres[2], A. Clark[1], S. Chripko[3], C. Deser[4], N. J. Dunstone[1], J. Garcìa-Serrano[5], G. Gastineau[6], L. S. Graff[7], S. C. Hardiman[1], B. He[8], L. Hermanson[1], T. Jung[9,10], J. Knight[1], X. Levine[11], G. Magnusdottir[12], E. Manzini[13], D. Matei[13], M. Mori[14], R. Msadek[3], P. Ortega[11], Y. Peings[12], A. A. Scaife[1,15], J. A. Screen[15], M. Seabrook[1], T. Semmler[9], M. Sigmond[16], J. Streffing[9], L. Sun[17], A. Walsh[15]

[1]Met Office Hadley Centre, FitzRoy Road, Exeter, UK

[2]Department of Meteorology, University of Reading, Reading, UK

[3]CECI, Université de Toulouse, CNRS, CERFACS, Toulouse, France

[4]National Center for Atmospheric Research, Boulder, CO, USA

[5]Group of Meteorology, Universitat de Barcelona, Barcelona, Spain

[6]UMR LOCEAN, Sorbonne Université /CNRS/IRD/MNHN, Institut Pierre Simon Laplace (IPSL), Paris, France

[7]Norwegian Meteorological Institute, Oslo, Norway

[8]State Key Laboratory of Numerical Modeling for Atmospheric Sciences and Geophysical Fluid Dynamics, Institute of Atmospheric Physics, Chinese Academy of Sciences, Beijing, China

[9]Alfred Wegener Institute, Helmholtz Centre for Polar and Marine Research, Bremerhaven, Germany

[10]Institute of Environmental Physics, University of Bremen, Bremen, Germany

[11]Barcelona Supercomputing Center, Jordi Girona 29 - 08034 Barcelona, Spain

[12]Department of Earth System Science, University of California Irvine, Irvine, CA, USA

[13]Max-Planck-Institut für Meteorologie, Bundesstraße 53, 20146 Hamburg, Germany

[14]Research Institute for Applied Mechanics, Kyushu University, Fukuoka, Japan

[15]College of Engineering, Mathematics and Physical Sciences, Exeter University, UK

[16]Canadian Centre for Climate Modelling and Analysis, Environment and Climate Change Canada, Victoria, British Columbia, Canada

[17]Department of atmospheric science, Colorado State University, Fort Collins, CO, USA

Corresponding author: Doug Smith, doug.smith@metoffice.gov.uk

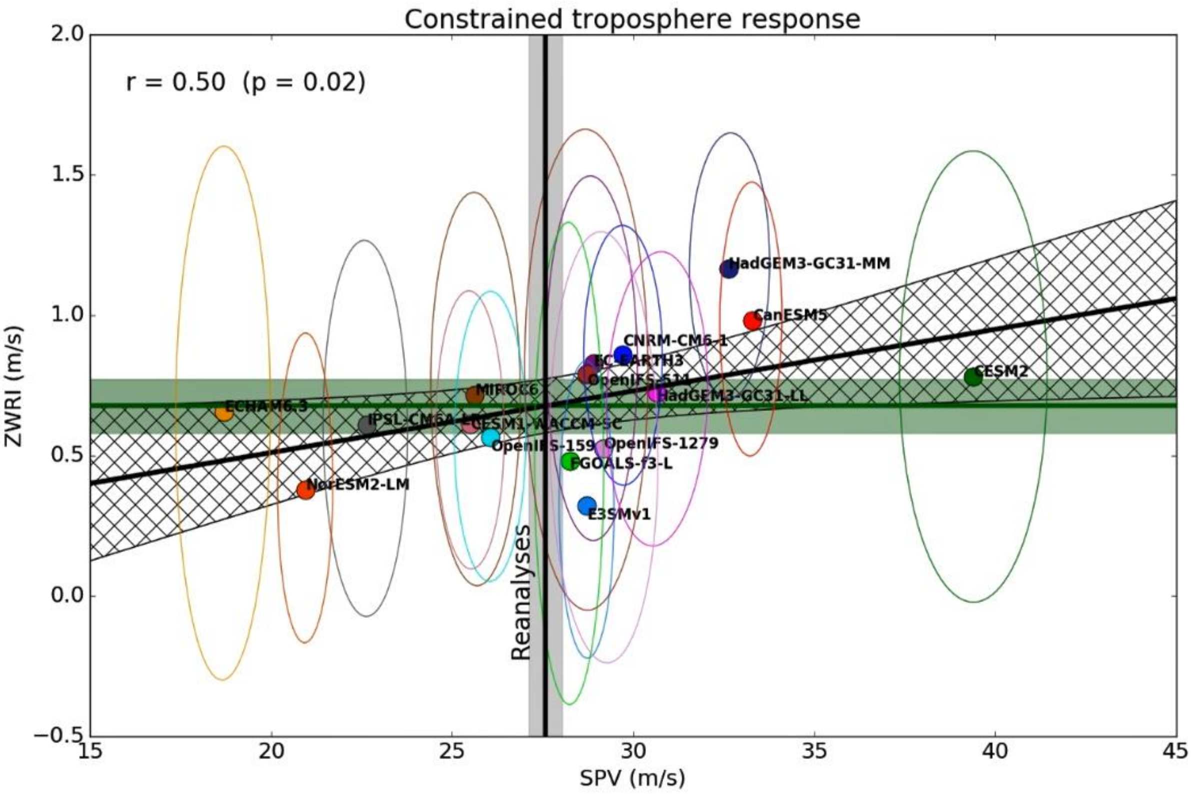

Supplementary Figure 1: *Emergent constraint based on the ensemble regression (ER) between background strength of stratosphere polar vortex (SPV) and the zonal wind response index (ZWRI). Black line shows the regression with hatching showing the 95% confidence interval. Horizontal green line shows the constrained ensemble mean response, with the shading showing its 95% confidence interval (Methods). Vertical black line and grey shading shows the mean and range of eddy feedback from the reanalyses. Ellipses show the 95% uncertainties obtained by bootstrapping with replacement the ensemble members. All data are for DJF.*
